# Obesity, Bariatric Surgery, and Cancer Risk: Nutritional Perspectives and Long-Term Clinical Implications

**DOI:** 10.3390/nu18040685

**Published:** 2026-02-20

**Authors:** Claudia Reytor-González, Gerardo Sarno, Martha Montalvan, Ludovica Verde, Giuseppe Annunziata, Luigi Barrea, Giovanna Muscogiuri, Daniel Simancas-Racines

**Affiliations:** 1 Facultad de Ciencias de la Salud y Bienestar Humano, Universidad Tecnológica Indoamérica, Ambato 180150, Ecuador; claudiareytor@gmail.com; 2 “San Giovanni di Dio e Ruggi D’Aragona” University Hospital, Scuola Medica Salernitana, 84131 Salerno, Italy; 3 Escuela de Medicina, Universidad Espíritu Santo, Samborondón 0901952, Ecuador; 4 Centro Italiano per la Cura e il Benessere del Paziente con Obesità (C.I.B.O.), Dipartimento di Medicina Clinica e Chirurgia, Unità di Endocrinologia, Diabetologia ed Andrologia, Università degli Studi di Napoli Federico II, 80131 Naples, Italy; 5 Division of Endocrinology, Department of Medicine, The University of Arizona College of Medicine, Tucson, AZ 85724, USA; 6 Department for the Promotion of Human Sciences and Quality of Life, San Raffaele Open University, 00166 Rome, Italy; giuseppe.annunziata@uniroma5.it; 7 Department of Psychology and Health Sciences, Centro Direzionale, Università Telematica Pegaso, 80143 Naples, Italy; 8 Dipartimento di Medicina Clinica e Chirurgia, Unità di Endocrinologia, Diabetologia ed Andrologia, Università degli Studi di Napoli Federico II, 80131 Naples, Italy; 9 Cattedra Unesco “Educazione Alla Salute E Allo Sviluppo Sostenibile”, Università degli Studi di Napoli Federico II, 80131 Naples, Italy

**Keywords:** obesity, cancer risk, bariatric surgery, inflammation, insulin resistance, micronutrient deficiencies, gut microbiota

## Abstract

Obesity is recognized as a causal risk factor for the development of multiple cancers, with risk magnitude varying by tumor site, sex, life stage, and adipose tissue distribution. This narrative review synthesizes recent epidemiological evidence linking excess body fatness with cancer incidence and mortality and integrates the biological mechanisms that explain this association. Chronic low-grade inflammation, insulin resistance with compensatory hyperinsulinemia, dysregulation of adipose-derived hormones and sex steroids, impairment of anti-tumor immune responses, alterations in the gut microbiota, and remodeling of the tumor microenvironment collectively create conditions that favor tumor initiation and progression. Bariatric surgery is the most effective clinical intervention for achieving substantial and sustained weight loss in individuals with severe obesity, and growing evidence indicates that it is associated with a reduction in overall cancer risk and cancer-related mortality, particularly for malignancies strongly linked to obesity. However, the extent of this benefit differs by surgical technique and remains less consistent for colorectal cancer. Beyond metabolic improvements, bariatric surgery produces long-term changes in nutritional physiology that may also influence oncologic outcomes. Persistent deficiencies of micronutrients such as iron, folate, vitamin B12, vitamin D, and calcium can affect DNA synthesis, methylation, oxidative balance, and cellular repair. Altered protein and energy intake may contribute to loss of lean mass and reduced metabolic resilience, while changes in alcohol absorption and metabolism can increase systemic exposure to ethanol and its carcinogenic metabolites. In addition, bariatric surgery induces sustained remodeling of the gut microbiome and bile acid metabolism, which may further modulate tumorigenic signaling. Overall, the oncological impact of bariatric surgery reflects a balance between metabolic improvement and long-term nutritional management, underscoring the need for structured follow-up and targeted nutritional strategies to optimize cancer risk reduction.

## 1. Introduction

Obesity has become one of the dominant public-health threats of the last few decades, with a steep rise in prevalence across most countries and age groups. The most recent Non-Communicable Disease Risk Factor Collaboration analysis (1990–2022) shows that obesity increased in the majority of countries worldwide, reflecting a sustained global shift toward higher adiposity rather than a transient epidemiologic fluctuation [1]. This matters far beyond weight itself: the health burden attributable to high body mass index (BMI) continues to grow globally, contributing to cardiometabolic disease, disability, and premature mortality [2,3]. In parallel, cancer remains a leading cause of death worldwide; updated global cancer statistics for 2022 estimate close to 20 million new cases and 9.7 million deaths, underscoring the scale of preventable risk factors that can realistically shift population-level outcomes [4,5].

Within this landscape, excess adiposity is now recognized as a causal risk factor for multiple cancer sites and a driver of cancer incidence and mortality trends in many settings. A key synthesis of the International Agency for Research on Cancer (IARC) evaluations highlighted that obesity is causally related to cancer at 13 anatomic sites, strengthening the argument that weight-related biology is not merely correlated with cancer risk but can sit upstream of carcinogenesis [6]. These findings are consistent with preventive models that highlight the combined influence of body composition, dietary quality, and physical activity as modifiable factors shaping cancer risk throughout life [7,8]. Importantly, obesity-related cancer risk is not uniform: the magnitude of association varies by cancer type, sex, menopausal status, metabolic phenotype, and exposure duration, suggesting that obesity represents a biologically heterogeneous condition that requires finer clinical characterization than BMI alone.

Mechanistically, multiple converging pathways plausibly connect obesity to tumor initiation and progression. Chronic low-grade inflammation and adipose tissue remodeling can alter the local tumor microenvironment (TME) and promote systemic pro-tumor signaling, positioning inflammation as a central and potentially reversible mediator of the obesity–cancer relationship [9]. Metabolic dysfunction—particularly insulin resistance and compensatory hyperinsulinemia—may further stimulate mitogenic and anti-apoptotic signaling via the insulin and insulin-like growth factor (IGF) axis, while also interacting with sex steroids and adipokines [10,11]. In parallel, adipokine dysregulation (e.g., leptin-adiponectin imbalance) and macrophage-driven inflammatory polarization can shape immune surveillance, angiogenesis, and cellular energetics in ways that favor tumorigenesis [12,13,14]. Collectively, these mechanisms underscore why nutritional status is clinically relevant, as diet quality, micronutrient adequacy, alcohol intake, and the gut microbiome can modulate inflammation, insulin sensitivity, sex hormone metabolism, and immune function—domains directly implicated in cancer risk biology.

Bariatric surgery (BS) represents the most effective long-term intervention for severe obesity, achieving sustained weight reduction and significant improvements in metabolic comorbidities [15]. Beyond weight reduction, surgery can improve glycemic trajectories and cardiometabolic risk through changes in gut hormones, bile acid (BA) signaling, nutrient flow, and food reward pathways—mechanisms that also intersect with inflammation and cancer-relevant biology [16]. However, bariatric procedures also impose long-term nutritional constraints: altered intake capacity, malabsorption (procedure-dependent), and changes in gastrointestinal physiology create a sustained risk of micronutrient deficiencies unless systematic monitoring and supplementation are implemented [17,18,19]. Major clinical guidance documents emphasize that micronutrient surveillance and individualized supplementation are not optional “add-ons” but core components of bariatric care [20,21,22].

Over the last decade, evidence has increasingly suggested that BS may be associated with a lower incidence of overall cancer and obesity-related cancers, as well as reductions in cancer mortality in some cohorts. Meta-analyses and large observational studies generally support a protective association. However, their effect sizes differ by cancer site, sex, and procedure type, and residual confounding remains a central limitation of non-randomized evidence [23,24,25,26]. At the same time, the story is not purely “surgery equals lower cancer risk”: BS can also introduce or amplify exposures relevant to cancer biology. One clinically important example is alcohol: after Roux-en-Y gastric bypass (RYGB), and in some contexts sleeve gastrectomy (SG), alcohol pharmacokinetics can change, and cohort evidence links surgery—particularly RYGB—with increased risk of alcohol use disorder–related hospitalizations, which may erode some long-term health gains [27]. Additionally, profound and procedure-specific shifts in the gut microbiome after BS have been repeatedly observed, raising questions about long-term consequences for inflammation, BA metabolism, and colorectal/gastrointestinal carcinogenesis pathways—areas where evidence is emerging but not yet definitive [28,29,30,31,32].

Taken together, these findings suggest that nutritional exposures may act as key mediators—rather than passive consequences—of cancer risk modification after BS. Cancer risk modulation in this context is likely shaped not only by reductions in adiposity, but also by long-term nutritional exposures, including micronutrient status, protein–energy balance, alcohol metabolism, diet quality, and surgery-induced changes in the gut microbiome—factors with established relevance to carcinogenesis, immune regulation, and metabolic signaling. Accordingly, the objective of this review is to critically examine the epidemiological evidence linking obesity and BS to cancer risk, and to integrate current knowledge on nutritional mechanisms and clinical nutritional management as key determinants of long-term oncological outcomes in post-bariatric patients.

## 2. Methods

This narrative review considered publications from database inception through December 2025. Literature searches were conducted in PubMed/MEDLINE, Cochrane Library, Scopus, and Google Scholar, using combinations of terms related to obesity, adiposity, body mass index, bariatric surgery, Roux-en-Y gastric bypass, sleeve gastrectomy, cancer risk, cancer incidence, cancer mortality, inflammation, insulin resistance, IGF-1, adipokines, sex hormones, gut microbiome, bile acids, micronutrient deficiencies, and alcohol metabolism.

Eligible publications included original observational studies, registry-based cohort studies, Mendelian randomization analyses, systematic and narrative reviews, meta-analyses, and relevant clinical guidelines examining associations between obesity, bariatric surgery, metabolic and nutritional alterations, and cancer risk or outcomes. Seminal articles published outside the predefined time frame were included when necessary to provide conceptual or mechanistic context.

Evidence was synthesized narratively by integrating epidemiological findings with biological and nutritional mechanisms to provide an interpretative overview of the field. Particular emphasis was placed on biological plausibility and consistency across epidemiological designs rather than on formal evidence grading, in alignment with the objectives of a narrative review. This review did not involve primary data collection or formal quantitative synthesis.

## 3. Obesity and Cancer: Epidemiological and Mechanistic Links

The association between obesity and cancer is no longer a question of whether a relationship exists, but rather how this relationship differs across cancer sites, populations, and biological contexts [33]. Contemporary evidence indicates that excess adiposity does not confer a uniform increase in cancer risk; instead, obesity acts as a context-dependent exposure, modifying cancer susceptibility through pathways that vary according to tissue type, sex, life stage, and the underlying metabolic phenotype. High-level syntheses from the IARC support causal associations between excess body fatness and multiple malignancies, providing a robust framework for interpreting site-specific patterns [34]. Recent advances in epidemiology, including refined anthropometric measures, long-term prospective cohorts, and Mendelian randomization (MR) approaches, have strengthened causal inference while simultaneously revealing important nuances. Tissue-partitioned MR, for example, supports the idea that cancer risk may depend not only on total adiposity but also on the biological component of adipose tissue relevant to endocrine and metabolic signaling, reinforcing the limitations of interpreting BMI as a stand-alone exposure [35].

### 3.1. Epidemiological Evidence Linking Obesity to Site-Specific Cancer Risk

Epidemiological studies conducted over the past decade consistently demonstrate that higher BMI is associated with increased risk across several major cancer sites, with clear dose–response gradients observed in large prospective cohorts. Importantly, these gradients are not uniform: effect sizes differ by cancer site, sex, and clinical context, reflecting distinct underlying biological mechanisms rather than random variability.

Among obesity-related malignancies, endometrial cancer (EC) exhibits one of the steepest and most consistent dose–response relationships with BMI [36]. Evidence from the Obesity and Disease Development Sweden (ODDS) pooled cohort, which included approximately 4.1 million individuals and evaluated more than 100 cancer forms and subtypes, quantified cancer risk using both categorical BMI comparisons (obesity vs. normal weight) and continuous BMI increments. Within this large population-based analysis, EC showed a hazard ratio (HR) of 1.68 per 5 kg/m^2^ increase in BMI, and women with obesity had a several-fold higher risk compared with those of normal weight [37]. This magnitude—especially for type I tumors—supports hormonally mediated pathways (notably estrogen exposure in the context of low progesterone) and metabolic inflammation, and it implies that modest shifts in population BMI distribution can materially change EC incidence [38].

In ODDS, Colorectal cancer (CRC) risk increased in a graded fashion with BMI. For colon adenocarcinoma, the HR per 5 kg/m^2^ higher BMI was 1.16 overall, with a higher estimate in men (1.24) than women (1.08). Rectal adenocarcinoma showed a smaller but still positive gradient (HR 1.09 per 5 kg/m^2^ overall) [37].

Another cohort study from the UK Biobank, including 460,784 participants followed for a median of 12.5 years, provides strong evidence that central obesity is a more relevant determinant of CRC risk than general obesity measured by BMI. In single-exposure models, each standard deviation increase in BMI, waist circumference, and waist-to-hip ratio (WHR) was associated with higher CRC risk, HR of 1.10 for BMI, 1.14 for waist circumference, and 1.18 for WHR, respectively. However, when BMI and WHR were mutually adjusted, the association with BMI was markedly attenuated (HR 1.04 per SD increase), whereas WHR remained robustly associated with CRC (HR 1.15 per SD increase). Notably, WHR predicted CRC risk across all BMI categories and in both sexes, whereas BMI showed weak or null associations after adjustment, particularly in women and for rectal cancer [39]. These findings indicate that visceral fat distribution accounts for a substantial proportion of obesity-related CRC risk, underscoring the importance of central adiposity as a key epidemiological and biological driver of colorectal carcinogenesis.

These contemporary cohorts estimate align with systematic evidence syntheses indicating that adult adiposity is a robust CRC risk factor, with effects that vary by subsite and sex—patterns that are hard to explain by screening behavior alone and support biological heterogeneity (e.g., BAs, insulin signaling, inflammation) across the colorectum [40].

For breast cancer (BC), the epidemiology is more nuanced than a single pooled estimate because associations differ by menopausal status and by the metabolic–vascular context. Multiple large prospective studies and dose–response meta-analyses indicate that higher BMI is associated with increased BC risk after menopause, whereas in premenopausal women the association is weak, null, or sometimes inverse, reflecting complex hormonal and metabolic interactions [41,42,43].

A prospective dose–response meta-analysis reported that each 5 kg/m^2^ increment in BMI was linked to a modest increase in overall BC risk (RR 1.02, 95% CI 1.01–1.04); however, stratified analyses indicated an inverse association in premenopausal women (SRR 0.98, 95% CI 0.96–0.99), in line with earlier evidence of menopausal status–specific effects [44]. More recent cohort evidence underscores the role of metabolic context: in pooled analyses from the European Prospective Investigation into Cancer and Nutrition and UK Biobank cohorts (~6793 postmenopausal cases), each 5 kg/m^2^ increment in BMI was more strongly associated with postmenopausal BC risk in women with cardiovascular disease (HR 1.31; 95% CI 1.16–1.47) than in women without (HR 1.13; 95% CI 1.11–1.16), suggesting that cardiometabolic health modifies the adiposity–BC link [45]. Beyond BMI, body composition offers additional insight. In women with normal BMI, a 2025 meta-analysis demonstrated that incremental increases in percent body fat were linked to greater postmenopausal BC risk (HR 1.19 per 5-unit increase; 95% CI 1.08–1.31), highlighting the role of fat mass and distribution beyond overall BMI [46].

Collectively, these data show that obesity significantly increases postmenopausal BC risk, with stronger associations in the presence of cardiometabolic comorbidity and with measures of adiposity beyond BMI. By contrast, in premenopausal women, the relationship is less consistent or inverse, possibly reflecting differences in endogenous estrogen exposure and other hormonal mechanisms across the life course [47].

For pancreatic cancer (PC), the direction of association is consistently positive in contemporary syntheses, but bias from pre-diagnostic weight loss can attenuate observed risks if not handled carefully (reverse causation). Mandic et al. highlight that cancer-associated weight loss may begin years before diagnosis and can therefore lead to underestimation of the BMI–PC association in conventional prospective analyses, underscoring the importance of lagged analyses and careful exclusion windows in epidemiologic designs [48]. Regarding quantitative evidence, one of the most robust pooled estimates derives from the Pancreatic Cancer Cohort Consortium, indicating that individuals in the highest compared with the lowest BMI quartile experienced a significantly increased risk of PC (adjusted OR 1.33; 95% CI 1.12–1.58; *p* < 0.001). In addition, central adiposity was particularly relevant among women, with the highest versus lowest WHR quartile showing a substantially elevated risk (adjusted OR 1.87; 95% CI 1.31–2.69; *p* = 0.003) [49]. More contemporary population-scale evidence from Asia also supports this association. Using population-level data from South Korea encompassing approximately 7.4 million individuals, analyses indicated that PC risk was higher among those with severe obesity (BMI ≥ 28 kg/m^2^) compared with individuals of normal weight (HR 1.16; 95% CI 1.11–1.23). Moreover, the combination of current smoking and BMI ≥ 25 kg/m^2^ was associated with a greater risk than that observed in never smokers with BMI < 23 kg/m^2^ (HR 1.55; 95% CI 1.46–1.65), highlighting how adiposity may interact with other exposures to amplify risk in real-world populations [50]. Finally, genetic approaches add a layer of causal inference on fat distribution. The MR analysis suggests abdominal adiposity, represented by WHR adjusted for BMI, may be more etiologically relevant than BMI alone for PC [51].

Beyond the major obesity-related malignancies, growing epidemiological evidence indicates that excess adiposity is associated with increased incidence of a broad spectrum of additional cancers. A comprehensive umbrella review and dose–response meta-analysis published in 2023 reported that obesity was significantly associated with a higher incidence of brain tumors, cervical cancer, kidney cancer, esophageal cancer (OEC), gastric cancer (GC), ovarian cancer, multiple myeloma, gallbladder cancer, bladder cancer, liver cancer, thyroid cancer, and Hodgkin’s lymphoma [33]. Importantly, dose–response analyses based on ten studies demonstrated that each 5 kg/m^2^ increase in BMI was associated with a 1.01- to 1.13-fold higher risk of general brain tumors, multiple myeloma, bladder cancer, and non-Hodgkin’s lymphoma, supporting a graded relationship rather than a purely categorical effect [33]. More detailed analyses using finer BMI increments showed steeper site-specific associations, with each 1 kg/m^2^ increase in BMI linked to a 6% higher risk of kidney cancer and a 4% higher risk of gallbladder cancer, underscoring the susceptibility of these malignancies to modest changes in adiposity [33].

Life-course analyses further extend these findings by implicating early adiposity exposure. A large meta-analysis focusing on early-life BMI reported that each 5 kg/m^2^ increase in early-life BMI was associated with a significantly higher risk of ovarian cancer in adulthood (RR 1.15; 95% CI 1.07–1.23), underscoring the importance of cumulative and temporally early adiposity in shaping long-term cancer susceptibility [38].

### 3.2. Mechanistic Pathways Linking Obesity to Carcinogenesis

**Figure 1:**
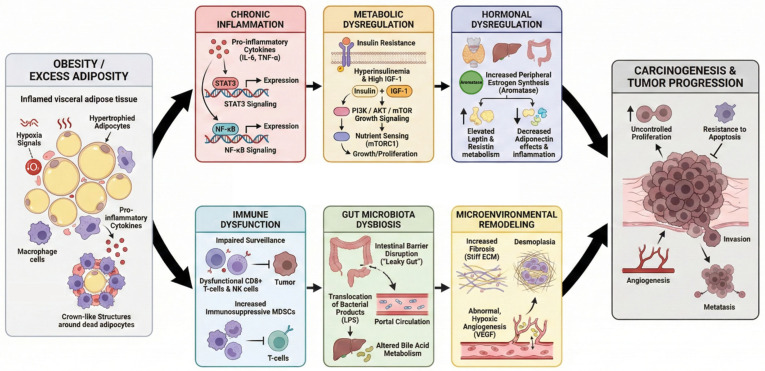
Mechanistic pathways linking obesity to carcinogenesis. Created in BioRender. Reytor, C. (2026) https://BioRender.com/xq8i7fg. Obesity promotes carcinogenesis through six interconnected mechanisms: (1) chronic inflammation [52,53,54,55,56,57,58], (2) metabolic dysfunction [59,60,61,62,63,64,65,66,67,68], (3) hormonal imbalance [69,70,71,72,73,74,75,76,77,78,79,80,81,82,83,84,85,86,87,88,89,90,91,92], (4) immune suppression [93,94,95,96,97,98,99], (5) gut microbiota alterations [100,101,102,103,104,105,106,107,108,109], and (6) remodeling of the tumor microenvironment [110,111,112,113,114,115,116,117,118]. Hypertrophic and hypoxic adipocytes drive inflammatory signaling and endocrine disruption [54,55,56,57,58,69,70,71,72,73,74,75,76]; systemic insulin resistance enhances mitogenic pathways; impaired immune surveillance favors tumor escape [93,94,95,96,97,98,99]; and microbiota-derived endotoxins sustain systemic inflammation [100,101,102,103,104,105,106]. Several of these pathways intersect with modifiable nutritional and metabolic exposures discussed throughout the manuscript, underscoring their relevance for long-term risk modulation after bariatric surgery. Abbreviations: STAT3: Signal Transducer and Activator of Transcription 3; NF-κB: Nuclear Factor kappa-B; IGF-1: Insulin-like Growth Factor 1; PI3K/AKT/mTOR: Phosphoinositide 3-kinase/Protein Kinase B/Mammalian Target of Rapamycin; NK cells: Natural Killer cells; MDSCs: Myeloid-Derived Suppressor Cells; LPS: Lipopolysaccharides; ECM: Extracellular Matrix; VEGF: Vascular Endothelial Growth Factor; TNF-α: Tumor Necrosis Factor alpha; IL-6: Interleukin 6; (↑) indicate increased expression, levels, or activity; (↓) indicate decreased expression, levels, or activity.

Obesity promotes carcinogenesis through a complex network of interrelated biological mechanisms that operate at systemic, tissue, and cellular levels. Rather than acting as a single causal exposure, excess adiposity creates a permissive biological environment characterized by chronic inflammation, metabolic dysregulation, endocrine perturbations, immune dysfunction, gut microbiota dysbiosis, and microenvironmental remodeling (Figure 1) [52,53]. The relative contribution of each pathway varies by cancer site, reflecting tissue-specific susceptibility and differences in hormonal, metabolic, and immunological context.

#### 3.2.1. Chronic Low-Grade Inflammation

Rather than representing a passive correlate of excess weight, obesity establishes a persistent state of low-grade chronic inflammation that reshapes tissue biology in ways that favor carcinogenesis. This process originates within expanding adipose tissue, where adipocyte hypertrophy generates hypoxia, endoplasmic reticulum stress, and mechanical strain. These stressors promote adipocyte turnover and cell death, triggering chemokine secretion and recruitment of innate and adaptive immune cells—particularly macrophages—into adipose depots, with visceral fat showing the most pronounced inflammatory remodeling [54]. As immune cells accumulate around stressed or dying adipocytes, crown-like structures form, and the local immune milieu shifts toward a pro-inflammatory phenotype characterized by sustained cytokine production [54,55,56]. Collectively, these alterations reflect a transition from adaptive immune activity to a chronic inflammatory state with recognized links to tumor biology.

This inflammatory response is not transient. Obesity alters immune-cell polarization and adipose tissue architecture in a manner that stabilizes inflammation over time, a process often described as metainflammation. Persistent immune activation is increasingly understood as a systemic exposure capable of preconditioning multiple tissues for malignant transformation rather than acting solely at tumor sites [57]. Mechanistic syntheses therefore conceptualize adipose inflammation as a mediator of obesity-associated cancer risk rather than a secondary epiphenomenon [58,119].

At the molecular level, obesity-related inflammation promotes carcinogenesis primarily through cytokine-driven oncogenic signaling. Interleukin-6 (IL-6) and tumor necrosis factor alpha (TNF-α) function as central hubs linking inflamed adipose tissue to epithelial, stromal, and immune reprogramming in distant organs [120,121]. Chronic IL-6 exposure activates the Janus kinase/signal transducer and activator of transcription 3 (JAK/STAT3) pathway, supporting proliferation, resistance to apoptosis, immune evasion, and metabolic adaptation, while TNF-α sustains inflammatory transcriptional programs via nuclear factor kappa B (NF-κB), promoting survival signaling and a pro-tumorigenic TME [122]. These convergent pathways provide a biologically coherent framework connecting systemic inflammation with cancer development.

Evidence from hepatocellular carcinoma (HCC) illustrates this inflammatory cascade. In both dietary and genetically induced obesity models, excess adiposity promotes hepatic inflammation and tumorigenesis through cytokine-dependent mechanisms converging on STAT3 signaling [123]. Downstream STAT3 activation induces angiogenic programs, including upregulation of vascular endothelial growth factor (VEGF), facilitating tumor vascularization and progression [53]. Within this context, chronic inflammation is interpreted as a mechanistic contributor to obesity-associated liver carcinogenesis, particularly where inflammatory signaling intersects with metabolic liver injury and fibrosis [124,125].

In the gastrointestinal tract, obesity-related inflammation similarly reshapes the colonic microenvironment [126,127]. Increased expression of inflammatory mediators—particularly TNF-α—interacts with canonical pathways in colorectal carcinogenesis, including Wingless-related integration site signaling, supporting the concept that inflammatory signaling may enhance epithelial susceptibility to malignant transformation [128,129,130,131]. Inflammatory pathways also interact with metabolic substrates, microbiota-derived mediators, and barrier dysfunction, helping explain why these mechanisms are most consistently linked to obesity-associated risk in HCC and CRC while also contributing to tumor progression in malignancies such as BC and EC [132,133].

#### 3.2.2. Metabolic Dysregulation

In obesity, insulin resistance (IR) in the liver, skeletal muscle, and adipose tissue prompts compensatory hyperinsulinemia to maintain euglycemia. This metabolic adaptation is increasingly implicated in carcinogenesis because insulin, beyond its metabolic role, can function as a growth-promoting signal capable of enhancing epithelial proliferation and influencing the TME. A key strength of recent literature is the analytical separation of insulin from glucose as causal candidates. In a large MR analysis, higher fasting insulin increased CRC risk (OR 1.65, 95% CI 1.15–2.36), whereas fasting glucose and 2-h glucose showed no clear association [59]. Chronic hyperinsulinemia can also bias insulin receptor signaling toward survival and proliferation programs in exposed tissues [60]. This divergence supports the interpretation that hyperinsulinemia itself may represent a biologically relevant exposure in colorectal tumorigenesis.

Hyperinsulinemia may further amplify mitogenic signaling through the insulin–IGF network by increasing the bioavailability of IGF-1 and activating overlapping downstream pathways [61]. Epidemiological support for this axis is notable due to convergence across serologic and genetic approaches. In UK Biobank analyses, higher circulating IGF-1 was associated with increased CRC risk (HR 1.24; 95% CI 1.10–1.40), with stronger estimates after broader biomarker adjustment (HR 1.34; 95% CI 1.18–1.52). Genetically predicted IGF-1 was similarly associated with CRC risk (OR 1.08; 95% CI 1.03–1.12) [62]. Together, these findings reinforce the biological plausibility of the insulin–IGF axis as a mediator linking metabolic dysfunction to cancer development.

At the signaling level, insulin and IGF-1 activate phosphoinositide 3-kinase (PI3K)/protein kinase B (AKT)/mechanistic target of rapamycin (mTOR) and mitogen-activated protein kinase (MAPK) pathways, which regulate proliferation, apoptosis resistance, and metabolic reprogramming—hallmarks of cancer when persistently upregulated [60,63]. In obesity, chronic nutrient excess sustains activation of these pathways, creating intracellular conditions favorable to tumor growth. Mechanistic syntheses consistently position mechanistic target of rapamycin complex 1 (mTORC1) as a central integrator of nutrient availability and growth-factor signaling in cancer biology [64,65,66].

Prolonged activation of mTORC1 under nutrient-rich conditions is associated with lowered thresholds for proliferative signaling and impaired cellular quality-control processes, offering a mechanistic explanation for how metabolic dysregulation may facilitate tumorigenesis [63,66]. Hyperinsulinemia also increases systemic substrate availability and interacts with hypoxic and inflammatory mediators, contributing to a microenvironment that supports tumor growth and immune evasion. Within this framework, metabolic dysregulation can be interpreted as a driver of tumor-supportive conditions rather than solely a metabolic disturbance.

Experimental data further contextualize these observations. In PC, a 2023 mechanistic study showed that hyperinsulinemia acting through acinar insulin receptors promoted early tumorigenic processes by increasing digestive enzyme production and local inflammation [67]. These findings provide experimental support for insulin signaling as an upstream contributor to pancreatic carcinogenesis.

These mechanisms do not operate uniformly across cancer types. Stronger associations tend to cluster in tissues characterized by prominent metabolic–endocrine signaling and exposure to portal or luminal nutrient flux, including the colorectum, liver, pancreas, endometrium, and postmenopausal breast tissue. However, causal inference remains complex, given heterogeneity in insulin measurement (fasting versus non-fasting assays) [59], potential horizontal pleiotropy in MR analyses, and the possibility of reverse causation in cancers with long preclinical phases, particularly PC. Not all genetic studies report concordant findings; one MR analysis observed no significant association between genetically predicted glycaemic traits and CRC risk, underscoring the need for cautious interpretation and methodological triangulation [68].

Metabolic pathways discussed here are therefore best understood as biologically plausible contributors to obesity-associated cancer risk, although the magnitude of their effect likely varies across tissues and metabolic contexts. BS induces sustained reductions in hyperinsulinemia and alters nutrient flux, positioning metabolic remodeling as one potential mechanism through which surgery may influence long-term cancer risk.

#### 3.2.3. Hormonal Dysregulation and Adipose-Derived Endocrine Signaling

Obesity reshapes the endocrine milieu through sustained changes in circulating hormones, receptor activation, and local paracrine microendocrine environments within adipose depots. Adipose tissue is not only an energy reservoir but an active endocrine and immunometabolic organ; when expanded and inflamed, it can amplify hormone-dependent signaling relevant to carcinogenesis, particularly in hormonally responsive sites such as the breast and endometrium [69,70,71]. This endocrine remodeling is increasingly interpreted as a mechanistic interface linking excess adiposity to tumor development.

##### Estrogen

Excess adiposity is linked to higher estrogen exposure, particularly through inflammatory remodeling of adipose tissue that increases local estrogen biosynthesis [72,73]. In postmenopausal women, adipose tissue becomes a key site of estrogen production, so obesity-related inflammation can translate into higher circulating and tissue estrogen levels even without ovarian contribution [74]. Concurrently, obesity is associated with lower sex hormone–binding globulin (SHBG), increasing the bioavailable fraction of estrogens and intensifying receptor-mediated signaling [75].

Weight-loss studies in postmenopausal women with overweight and obesity have demonstrated shifts toward less estrogenic hormonal profiles, supporting the dynamic nature of this endocrine environment [76]. Sustained estrogen signaling promotes proliferation and survival programs in hormone-sensitive tissues through pathways such as PI3K/AKT and MAPK/ERK [70,72,77,78,79]. Beyond receptor-mediated effects, estrogen metabolism can generate reactive intermediates capable of forming DNA adducts and contributing to mutagenic damage, strengthening biological plausibility for DNA-damage mechanisms in obesity-related breast carcinogenesis [80,81,82].

However, estrogen biology is tissue- and receptor-context dependent. In the colon, estrogen receptor beta predominates and has been linked to protective signaling, including differentiation, inflammation control, and enhanced DNA repair capacity [83]. Similarly, experimental work in obesity-related prostate settings suggests estrogenic signaling may attenuate inflammatory remodeling in periprostatic adipose tissue under high-fat feeding [84]. These observations underscore the context-specific effects of estrogen signaling across tissues.

##### Progesterone

Progesterone counterbalances estrogen-driven endometrial proliferation by promoting differentiation and limiting sustained mitogenic signaling [85]. When ovulation is infrequent or absent, as commonly observed in polycystic ovary syndrome coexisting with obesity, prolonged estrogen exposure without progesterone opposition increases the likelihood of endometrial hyperplasia and progression toward EC [86,87]. Many endometrial neoplasms retain hormone sensitivity, and higher progesterone receptor expression has been associated with a more favorable prognosis [88].

A key limitation is progestin resistance. Activation of the PI3K/AKT pathway—frequent in endometrial carcinogenesis—can impair progesterone receptor function and reduce progesterone responsiveness, providing a mechanistic explanation for variable therapeutic responses [85,89,90]. Observational data evaluating local progestin delivery via a levonorgestrel intrauterine system suggest differential clinical outcomes compared with systemic administration, although interpretation remains constrained by study design and population heterogeneity [91,92].

##### Leptin

Leptin concentrations increase in proportion to fat mass and remain chronically elevated in obesity. Beyond its role in appetite regulation, leptin exerts pro-oncogenic effects by activating signaling pathways involved in cancer development, including JAK/STAT3, PI3K/AKT, and MAPK cascades [134]. These pathways promote proliferation, survival, migration, and resistance to apoptosis across multiple cancer models [12,135].

Leptin also functions as a pro-angiogenic mediator by inducing VEGF expression and interacting with hypoxia-driven signaling, thereby facilitating tumor vascularization and growth in obesity-associated cancers. Experimental and translational evidence indicate that leptin signaling enhances angiogenic processes within the TME, particularly in BC, where activation of VEGF-related pathways supports neovascularization and tumor progression [136].

In hormone-dependent malignancies, leptin further amplifies carcinogenic signaling through molecular crosstalk with estrogen receptor pathways. Experimental evidence shows that leptin enhances estrogen-mediated transcriptional activity and proliferative responses in estrogen receptor–positive BC cells, whereas inhibition of estrogen receptor signaling attenuates leptin-induced cell growth [137]. This functional interdependence supports leptin as a contributor to hormonally mediated tumor biology.

##### Adiponectin

Adiponectin is an adipocyte-secreted hormone whose circulating levels decrease with increasing adiposity and insulin resistance. Mechanistically, adiponectin engages its receptors (AdipoR1/R2) to activate AMP-activated protein kinase, with downstream signaling capable of suppressing pro-growth pathways such as PI3K/AKT/mTOR and MAPK/ERK. These effects are associated with reduced proliferation and enhanced metabolic regulation in cancer-prone cells [138].

Adiponectin also modulates inflammatory signaling within the TME. Experimental studies show diminished NF-κB and STAT3 activation following adiponectin exposure, supporting a role in counteracting chronic inflammation linked to carcinogenesis and metastatic progression [139]. Additionally, adiponectin influences immune regulation, including macrophage polarization toward anti-inflammatory phenotypes and reduced macrophage infiltration in adipose tissue [140]. Collectively, these mechanisms position adiponectin as a potential protective component within the adipokine milieu.

##### Resistin

Resistin, primarily secreted by immune cells within adipose tissue in humans, is elevated in obesity and closely linked to chronic inflammation and insulin resistance. Increasing evidence implicates resistin in cancer biology through activation of pro-inflammatory and pro-tumorigenic pathways, including Toll-like receptor 4 (TLR4) and NF-κB signaling. These pathways enhance cancer cell proliferation, invasion, and metastatic potential while reinforcing a permissive inflammatory TME [141]. Clinically, higher circulating resistin levels have been associated with poorer prognosis and more aggressive phenotypes in several obesity-related malignancies, supporting its role as a mediator rather than a passive marker of cancer risk [142].

##### Novel Adipokines

Beyond classical adipokines, emerging mediators such as visfatin, chemerin, omentin, and vaspin are increasingly recognized as contributors to obesity-associated carcinogenesis. Visfatin has been linked to enhanced inflammation, angiogenesis, and metabolic reprogramming in tumor cells, whereas chemerin and omentin appear to modulate immune cell recruitment and inflammatory tone within adipose tissue and the tumor microenvironment. Although their roles remain incompletely characterized, accumulating translational evidence associates altered adipokine profiles with cancer incidence and progression [143].

#### 3.2.4. Immune Dysfunction

Beyond inflammation, obesity compromises anti-tumor immune surveillance by impairing the function of cytotoxic effector cells and antigen-presenting compartments within the TME [93]. This disruption reflects a broader immunometabolic shift in which nutrient excess and lipid accumulation reshape immune-cell behavior toward tumor tolerance. CD8^+^ T-cell dysfunction has been consistently described in obesity-associated tumor environments, where altered nutrient availability and lipid-rich conditions are associated with impaired cytotoxic programs and may facilitate tumor progression [93]. Immunometabolic syntheses further indicate that obesity remodels CD8^+^ T-cell metabolism, potentially reducing effector fitness and influencing responses to T cell–based immunotherapies [94].

Experimental evidence suggests that intratumoral T-cell dysfunction may exhibit biological plasticity, as metabolic reprogramming has been associated with partial restoration of CD8^+^ T-cell activity in preclinical models [95]. Obesity has also been linked to alterations in natural killer (NK) cell immunity, including reduced cytotoxic capacity that could weaken early tumor control [96]. Mechanistic studies further suggest that lipid excess may directly suppress NK-cell activity, providing a plausible pathway for diminished innate tumor surveillance [97].

In parallel, obesity appears to favor expansion and tumor recruitment of immunosuppressive myeloid populations, particularly myeloid-derived suppressor cells (MDSCs), which dampen T-cell function and support immune escape [98]. Evidence also indicates that obesity may impair antigen presentation; models of diet-induced obesity demonstrate disruption of dendritic-cell metabolic and functional programs, potentially undermining the initiation and maintenance of effective anti-tumor responses [96,99]. Collectively, CD8^+^ T-cell dysfunction, NK-cell impairment, MDSC expansion, and altered dendritic-cell priming point toward a coordinated shift that may contribute to an immune-permissive microenvironment in obesity-associated cancers [93].

It is important to recognize that several mechanistic insights described above derive primarily from experimental and preclinical models. While these findings provide biologically plausible frameworks linking obesity-related immune dysfunction to tumor progression, direct confirmation in long-term human studies remains limited. Accordingly, these pathways should be interpreted as mechanistic hypotheses rather than definitive causal relationships.

#### 3.2.5. Gut Microbiota Dysbiosis

Gut microbiota dysbiosis increases cancer risk by disrupting epithelial barrier integrity, reshaping microbial metabolite profiles, and amplifying host–microbe inflammatory signaling, particularly along the gut–liver axis [100,101]. These links are strongest for CRC and HCC, where mechanistic pathways are consistently synthesized across experimental and translational literature [29,30,102]. Collectively, dysbiosis is increasingly viewed as a biologically plausible mediator connecting obesity to site-specific carcinogenesis.

A central mechanism involves gut barrier dysfunction characterized by increased intestinal permeability. This facilitates translocation of microbial products, including lipopolysaccharide, into the circulation—often via the portal vein—activating pattern-recognition receptor signaling and reinforcing tumor-permissive inflammatory programs [103]. Dysbiosis also alters production of short-chain fatty acids (SCFAs) such as butyrate, which normally support epithelial homeostasis and immunoregulatory signaling [104]. Altered SCFA availability has been associated with disrupted epithelial regulation and loss of protective metabolic signaling in CRC models [105].

In parallel, dysbiosis reshapes BA metabolism, increasing exposure to secondary BAs with pro-tumorigenic properties mediated through oxidative stress, DNA damage signaling, and microenvironmental remodeling [106]. These mechanisms are most directly relevant to CRC and HCC, where local exposure and portal circulation provide biologically coherent routes of carcinogenesis [102,103]. Evidence in other cancers, including pancreatic ductal adenocarcinoma and GC, remains emerging and heterogeneous, suggesting that the oncogenic implications of microbiota alterations may be context dependent [107,108,109].

#### 3.2.6. Microenvironmental Remodeling

Obesity can promote carcinogenesis by restructuring local tissue ecosystems through coordinated changes in stromal composition, extracellular matrix (ECM) architecture, and vascular function [110,111]. ECM remodeling and interstitial fibrosis are consistent hallmarks of obesity, leading to increased stiffness and altered mechanotransduction that favor tumor-supportive phenotypes [112,113,114,115]. These structural alterations contribute to a microenvironment increasingly permissive to tumor initiation and progression.

Obesity also induces desmoplasia and vascular dysfunction. Experimental models demonstrate that obesity-driven stromal remodeling is accompanied by impaired perfusion and hypoxic stress, reinforcing tumor progression beyond systemic metabolic effects [110]. Angiogenic remodeling further contributes, with obesity supporting abnormal vasculature associated with aggressive tumor behavior and therapy resistance [116,117]. Together, these features position stromal remodeling as a local amplifier of carcinogenic signaling.

Finally, obesity alters stromal cellular composition through adipose-derived progenitors. Obesity-activated adipose-derived stromal/stem cells enhance mammary tumor growth and invasion, providing a direct local mechanism through which adipose niches may facilitate tumor progression [56,118]. Microenvironmental remodeling, therefore, reflects the integration of mechanical, vascular, and cellular alterations that collectively support tumor development within obesity-altered tissues.

Overall, the mechanistic links between obesity and carcinogenesis reflect a convergence of systemic exposures and local tissue-level remodeling (Table 1). Chronic inflammation, metabolic dysfunction, hormonal imbalance, immune suppression, gut microbiota alterations, and microenvironmental remodeling interact to create site-specific permissive conditions for tumor initiation and progression [52,53,54,55,56,57,58,59,60,61,62,63,64,65,66,67,68,69,70,71,72,73,74,75,76,77,78,79,80,81,82,83,84,85,86,87,88,89,90,91,92,93,94,95,96,97,98,99,100,101,102,103,104,105,106,107,108,109,110,111,112,113,114,115,116,117,118,119,120,121,122,123,124,125,126,127,128,129,130,131,132,133,134,135,136,137,138,139,140,141,142,143]. This integrative framework provides a biological context for interpreting how weight loss and BS may help explain variations in cancer risk.

### 3.3. Role of Dietary Factors and Nutrient Imbalances in Mediating These Risks

Dietary exposures can plausibly mediate or modify obesity-associated cancer risk because they act on the same upstream pathways highlighted in Section 3.2—chronic low-grade inflammation, insulin–growth factor signaling, oxidative stress, BA metabolism, and hormonal regulation—often independently of BMI [160,162].

In obesity, diet is therefore best conceptualized as a biologically active co-determinant, and not just a correlate of higher body mass, that can amplify or attenuate carcinogenic signaling across tissues [144,159,163,164].

A central and clinically actionable dimension is overall diet quality and the degree of industrial processing. In a large UK Biobank cohort analysis evaluating ultra-processed food (UPF) intake against multiple site-specific cancers and cancer mortality, higher UPF consumption was associated with higher risks for several cancer outcomes and cancer-related mortality, although associations varied by cancer site [145,146]. A 2024 systematic review and meta-analysis focused on gastrointestinal cancers similarly reported that higher UPF intake was associated with increased risk of CRC (HR 1.11; 95% CI 1.03–1.21; *p* = 0.01; *I*^2^ = 31%), colon cancer (HR 1.12; 95% CI 1.02–1.23; *p* = 0.02; *I*^2^ = 0%), and non-cardia gastric cancer (HR 1.43; 95% CI 1.02–2.00; *p* = 0.04; *I*^2^ = 0%), while associations for other gastrointestinal sites were not consistently observed, supporting heterogeneity by anatomical site and underlying mechanism [147]. This pattern is biologically coherent in obesity, where UPF-heavy diets typically combine high energy density and refined carbohydrates with low fiber and higher sodium/additive exposure, features linked to postprandial glycemic excursions, inflammatory tone, and gut barrier perturbation [148,149].

For CRC, the evidence base linking specific dietary components to risk is particularly mature. The World Cancer Research Fund and the American Institute for Cancer Research Continuous Update Project have judged the evidence convincing that processed meat and alcoholic drinks increase CRC risk and that foods containing wholegrains and dietary fiber protect against CRC [150]. Recent meta-analytic evidence continues to support positive associations between higher red/processed meat consumption and CRC incidence [151,152], reinforcing guidance to limit these exposures as part of cancer prevention strategies. Mechanistically, these diet–cancer links align with obesity-related pathways through inflammation, insulin signaling, and microbiome-mediated BA metabolism, helping explain why colorectal carcinogenesis is especially sensitive to diet composition in settings of excess adiposity [153,154].

Alcohol deserves separate emphasis because it acts as a carcinogenic exposure with dose–response evidence in multiple cancers and can synergize with obesity-related endocrine and inflammatory signaling. A 2024 systematic review synthesizing evidence up to late 2023 reiterates that alcohol is an established cause of female BC and details consistent risk increases with higher intake across subgroups [157]. In addition, authoritative public health syntheses emphasize that alcohol causally increases risk for several cancers, including BC and CRC, with risk rising as consumption increases and signals detectable even at relatively low consumption levels in some cancer types [155]. In obesity, where estrogenic and inflammatory pathways may already be upregulated, alcohol exposure is particularly relevant to address as a modifiable co-factor in overall risk reduction [156].

Beyond dietary factors, obesity is frequently accompanied by clinically meaningful nutrient imbalances—a phenomenon sometimes described as paradoxical malnutrition (energy excess with micronutrient inadequacy) [165,166]. Narrative syntheses and clinical studies document a higher prevalence of deficiencies in vitamin D, folate, vitamin B12, iron, and other micronutrients among individuals with obesity, driven by dietary patterns, inflammation-related sequestration, and altered distribution/metabolism of fat-soluble vitamins [19,167,168,169,170,171,172]. Importantly, these imbalances are often present before BS: pre-operative assessments in patients with severe obesity seeking bariatric procedures report high rates of micronutrient deficiencies—particularly vitamin D deficiency/insufficiency and notable proportions with folate and vitamin B12 deficits—supporting routine screening as part of baseline risk characterization [169,171].

These imbalances matter for carcinogenesis because micronutrients contribute to DNA synthesis and repair, redox balance, methylation reactions, and immune competence. Folate illustrates the nuance required: a recent systematic review and meta-analysis suggests that higher dietary and total folate intake may be associated with lower CRC risk in primary prevention, while also highlighting biological concerns that folic acid supplementation could have different effects depending on timing and whether neoplastic foci already exist [173].

Vitamin D is another high-interest exposure: a 2024 umbrella review reports mostly inverse associations between circulating 25-hydroxyvitamin D and cancer incidence and mortality across reviewed outcomes, while appropriately cautioning that much of the evidence is observational and causality cannot be assumed [174]. In severe obesity, where vitamin D deficiency is common and inflammation is prevalent, these observations strengthen the rationale for careful assessment and correction of deficiencies as part of long-term risk management, even if supplementation is not a stand-alone anticancer intervention [175,176,177,178].

Finally, dietary exposures can be integrated at the pattern level through indices that approximate inflammatory potential. Meta-analytic evidence indicates that higher Dietary Inflammatory Index scores are associated with higher CRC risk, consistent with the role of chronic inflammation as a mediator of obesity-associated cancer susceptibility [148,154]. This is relevant clinically because inflammatory dietary profiles tend to cluster with central adiposity and insulin resistance, suggesting that dietary change may reduce risk not only by supporting weight control but also by directly shifting inflammatory signaling.

Overall, the evidence supports a dual framing: (i) dietary factors such as UPF intake, processed meat, low fiber or wholegrain consumption, and alcohol can directly influence cancer risk through pathways central to obesity-related carcinogenesis, including chronic inflammation, insulin–IGF signaling, hormonal dysregulation, and gut-related mechanisms [147,150,157], and (ii) nutrient imbalances, particularly deficiencies in vitamin D, iron, folate, and vitamin B12, are common in severe obesity and may intersect with immune regulation, oxidative stress, and genomic stability, plausibly shaping long-term cancer susceptibility in a site-specific manner [169,174,179]. These relationships are summarized in Table 2.

## 4. BS and Cancer Risk: Evidence and Controversies

BS has moved from being viewed solely as a weight-loss intervention to a plausible cancer-risk–modifying strategy because it produces durable improvements in adiposity, IR, and obesity-related inflammation, pathways that are causally linked to several malignancies [26]. Although the human evidence base remains dominated by observational and cohort studies, it is now sufficiently mature to support a nuanced interpretation: overall cancer incidence and cancer-related mortality tend to decrease after BS, particularly for obesity-associated cancers, while important site-specific, procedure-specific, and population-specific heterogeneity persists [180,181,182]. Consequently, despite more than two decades of epidemiological research, the relationship between BS and cancer risk remains complex and, in some contexts, controversial, warranting careful consideration of study design, comparator choice, and biological plausibility.

Taken together, current evidence supports a generally protective association between BS and overall cancer outcomes; however, the magnitude and direction of this effect vary considerably across studies. Much of this variability appears to be driven by methodological differences—including comparator selection, follow-up duration, and baseline patient risk—highlighting the need for cautious, context-aware interpretation of post-surgical cancer estimates.

### 4.1. Overall Cancer Incidence and Cancer-Related Mortality After BS

Evidence linking BS to cancer outcomes derives almost exclusively from observational cohorts and registry-based studies, as no randomized trials have been designed with cancer incidence as a primary endpoint. As a result, interpretation relies heavily on study design, comparator selection, and duration of follow-up.

Early retrospective cohort studies suggested a protective association between BS and cancer risk. In a cohort of 9949 patients undergoing RYGB, Adams et al. (2007) observed a significant reduction in cancer-related mortality compared with matched nonsurgical controls (HR 0.60; 95% CI 0.45–0.67), with stronger effects among women, supporting a potential role of BS in modifying long-term cancer risk [183].

Shortly thereafter, Christou et al. (2008) reported a markedly lower incidence of overall cancer in surgically treated patients with obesity compared with non-surgically treated controls in a matched cohort of 1035 individuals (RR 0.22; 95% CI 0.14–0.35), suggesting sustained weight loss as a key mediator of reduced cancer susceptibility [184]. Prospective evidence was subsequently provided by the Swedish Obese Subjects study, in which Sjöström et al. (2009) demonstrated a lower overall cancer incidence among surgically treated participants compared with conventionally treated individuals with obesity controls (HR 0.67; 95% CI 0.53–0.85), along with reduced cancer-related mortality during long-term follow-up, although cancer outcomes were secondary endpoints [185].

In contrast, Östlund et al. (2010) reported no significant reduction in overall cancer risk when BS patients were compared with the general population (SIR 1.04; 95% CI 0.93–1.17), highlighting how comparator choice can materially influence estimates of cancer risk after BS [186]. Concerns regarding site-specific risk were further amplified by Derogar et al. (2013) [187], who analyzed a retrospective cohort of 15,095 BS patients and 62,016 matched controls. The authors reported an increased incidence of CRC in the surgical cohort (overall SIR 1.60; 95% CI 1.25–2.02), with risk increasing over longer follow-up (SIR 2.00; 95% CI 1.48–2.64), challenging assumptions of uniform cancer risk reduction after BS [187].

Overall, these early divergent findings illustrate how comparator choice critically shapes risk interpretation. Comparisons with nonsurgical populations with obesity tend to suggest protective associations, whereas comparisons with the general population frequently attenuate or neutralize these effects, underscoring the methodological sensitivity of post-bariatric cancer estimates.

More contemporary evidence using non-surgical comparators with obesity has yielded more favorable estimates. Schauer et al. (2019) examined 22,198 patients undergoing BS matched to 66,427 non-surgical patients with severe obesity and reported a 33% lower hazard of developing any cancer (HR 0.67; 95% CI 0.60–0.74), with particularly strong associations for obesity-associated cancers such as postmenopausal breast, endometrial, colon, and pancreatic cancer [188]. Consistent procedure- and site-specific patterns were also observed in national administrative data from England, where a population-based cohort study (2019) found that BS was associated with reduced risk of hormone-related cancers, while gastric bypass was linked to an increased risk of CRC [189].

Convergent findings come from large, well-matched cohort data with extended follow-up. In a matched cohort study published by Aminian et al. (2022), BS was associated with a lower risk of incident obesity-associated cancer and reduced cancer-related mortality compared with nonsurgical care; the 10-year cumulative incidence of cancer mortality was 0.8% versus 1.4%, corresponding to an adjusted HR of 0.52 for cancer death [24]. Similarly, in a very large matched cohort, Lazzati et al. (2023) [190] analyzed 303,709 patients who underwent BS and 605,140 matched nonsurgical controls. BS was associated with a significantly lower hazard of overall cancer incidence (HR 0.76; 95% CI 0.59–0.98) and lower overall mortality (HR 0.60; 95% CI 0.56–0.64), with lower incidence rates of esophagogastric cancer despite small absolute event numbers [190].

Importantly, the magnitude of association appears to depend on patient context, including baseline risk and remaining latency window. In a 2024 cohort focused on adults aged ≥60 years, BS as a composite exposure was not consistently associated with lower rates of obesity-related cancer, although gastric bypass showed evidence of reduced risk versus matched controls. These findings support the notion that cancer-preventive benefits of BS may attenuate with older age or shorter follow-up horizons [178].

More recent registry-based evidence has introduced further nuance. Stenberg et al. (2025) [191] evaluated a nationwide Swedish cohort of 68,424 patients undergoing BS matched to 640,944 general population controls. No meaningful difference in overall cancer incidence was observed between groups (IRR 1.03; 95% CI 0.99–1.07). While reduced risks were observed for BC in women and skin cancers in both sexes, increased risks persisted for colon, liver, pancreatic, endometrial, renal cancers, malignant meningioma, and non-Hodgkin lymphoma [191]. Emerging evidence suggests that procedure-specific factors may also contribute to hepatic risk; notably, RYGB has been associated with progression of liver fibrosis in susceptible individuals, a biologically plausible pathway toward HCC, independent of alcohol-related mechanisms.

Collectively, contemporary cohort data suggest that BS is associated with lower overall cancer risk primarily when evaluated against untreated severe obesity, yet this protective signal becomes less consistent in general population comparisons. These patterns indicate that residual obesity-related risk, surveillance differences, and population baseline characteristics likely contribute to observed heterogeneity.

Evidence derived from systematic reviews and meta-analyses further underscores the heterogeneity of the association between BS and cancer risk. Early syntheses highlighted substantial data limitations; notably, Scozzari et al. (2013) concluded that the incidence of esophagogastric cancer after BS could not be reliably estimated due to limited case numbers and insufficient follow-up [192]. By contrast, a meta-analysis by Tee et al. (2013) [193], including four prospective and two retrospective studies, found an overall reduction in cancer risk after BS (RR 0.55; 95% CI 0.41–0.73). This association was significant among women (RR 0.68; 95% CI 0.60–0.77) but not among men (RR 0.99; 95% CI 0.74–1.32), underscoring sex-specific heterogeneity [193].

Subsequent large-scale meta-analyses have generally supported a reduction in overall cancer incidence after BS, while consistently emphasizing substantial between-study variability [194]. A meta-analysis published in 2024 synthesizing data from 33 cohort studies concluded that BS was associated with a significant reduction in overall cancer incidence, with protective associations observed for both gastric bypass and SG in subgroup analyses [24], in agreement with similar reports [26,194,195]. Site-specific meta-analyses suggest potential protective effects for PC [196] and for breast, endometrial, and ovarian cancers in women with obesity [158], whereas evidence for CRC remains inconsistent [180]. Importantly, a dedicated meta-analysis focusing on early-onset colorectal cancer (EOCRC) failed to demonstrate a significant impact of BS on EOCRC risk, underscoring persistent uncertainty for this cancer site [197].

Taken together, the persistent inconsistency observed for colorectal cancer likely reflects multifactorial influences, including latency effects, procedure-related physiological changes, differences in screening practices, and residual metabolic risk. Importantly, much of the observed discordance appears to arise from methodological and biological heterogeneity across studies. Comparisons with the general population may overestimate post-surgical risk by contrasting metabolically high-risk patients with baseline population averages, whereas studies using individuals with severe obesity as comparators more often suggest neutral or protective associations. Follow-up duration further contributes to variability, as colorectal carcinogenesis involves long latency windows that may not be adequately captured in shorter observational periods. Procedure type also warrants consideration, particularly for RYGB, which induces marked alterations in BA circulation, microbial composition, and luminal metabolites—including compounds such as tyramine—that may influence colonic epithelial exposure over time. Collectively, these factors suggest that discrepant CRC findings are more plausibly explained by differences in study design, comparator selection, surgical physiology, and microbiome-related exposures rather than by a uniform carcinogenic or protective effect of BS itself. Therefore, current evidence supports a context-dependent interpretation of CRC risk following BS rather than a single directional conclusion.

### 4.2. Procedure-Specific Effects: RYGB vs. SG vs. Adjustable Gastric Banding (AGB) vs. Biliopancreatic Diversion with Duodenal Switch (BPD-DS)

Procedure type is mechanistically relevant due to marked differences in malabsorption, BA flux, gut microbiome remodeling, and long-term micronutrient trajectories. These differences may therefore also be translated into heterogeneous cancer risk profiles across bariatric procedures. However, direct comparative evidence on cancer risk across bariatric procedures remains substantially thinner than that available for cardiometabolic outcomes. A large registry–cancer registry linkage study comparing RYGB, SG, AGB, and BPD-DS exemplifies the field’s shift from binary “surgery vs. no surgery” comparisons toward procedure-stratified cancer incidence analyses [198].

Overall, the emerging procedure-stratified literature suggests that cancer risk after BS is unlikely to be uniform across surgical approaches. Instead, procedure-specific physiological alterations may interact with baseline metabolic risk, emphasizing the need to interpret post-surgical cancer patterns within a mechanistic and procedural context rather than treating BS as a homogeneous exposure.

Where comparative evidence exists for CRC specifically, recent syntheses suggest that both RYGB and SG are associated with lower CRC risk compared with non-surgical comparators in several datasets. By contrast, direct head-to-head differences between RYGB and SG are often not statistically significant in pooled analyses, highlighting the limits of current comparative resolution [180,199].

This apparent paradox—protective associations relative to severe obesity but limited differentiation between procedures—likely reflects insufficient statistical power, heterogeneous follow-up durations, and residual confounding rather than true equivalence of biological effect.

A newer and clinically important line of evidence compares post-surgical cancer patterns with those of the general population. This design can reveal paradoxical “excess risk” signals, even when surgery appears beneficial relative to usual care in obesity. In a 2025 nationwide analysis, procedure-stratified signals—such as increases for certain cancer sites after RYGB—were observed. The authors emphasized that these findings depend strongly on comparator choice and warrant cautious interpretation and further confirmation [191]. Importantly, comparisons with the general population may inflate apparent post-surgical risk because they contrast metabolically high-risk individuals with baseline population averages, reinforcing comparator selection as a major source of heterogeneity in procedure-level analyses.

Direct procedure-to-procedure comparisons remain scarce. In a large registry–cancer registry linkage study with long-term follow-up, AGB (HR 1.26, *p* = 0.03) and BPD-DS (HR 1.91, *p* < 0.01) were associated with a significantly higher hazard of incident cancer compared with RYGB. By contrast, SG did not differ significantly from RYGB (HR 1.17, *p* = 0.33) [198].

Taken together, available data tentatively position RYGB and SG as having comparable oncologic profiles, whereas more malabsorptive procedures may carry distinct long-term risk trajectories; however, the observational nature of these comparisons precludes definitive causal interpretation.

Evidence for CRC, the most debated site, further illustrates these uncertainties. While some national cohort data have raised concerns that gastric bypass might be associated with higher CRC incidence compared with other approaches—particularly when comparisons are made with the general population [189]—other population-level analyses suggest that CRC risk after BS is similar to, or lower than, that of the general population and clearly lower than in individuals with untreated severe obesity [200]. Taken together, these discrepant findings likely reflect differences in comparator selection, follow-up duration, surveillance intensity, and baseline obesity-related risk rather than consistent procedure-specific carcinogenic effects. Therefore, the current body of evidence does not support a uniform procedure-driven increase in CRC risk but instead points toward context-dependent estimates shaped by study design and population characteristics.

In summary, procedure-specific cancer risk after BS remains incompletely characterized. Divergent findings across studies are most plausibly explained by methodological variability, comparator choice, and differences in patient baseline risk rather than clear biologically deterministic effects of any single procedure. Future studies with longer follow-up and refined causal designs will be essential to clarify whether true procedure-dependent oncologic trajectories exist.

### 4.3. Sex- and Age-Specific Patterns

Accumulating evidence indicates that the association between BS and cancer risk is modified by both sex and age, suggesting that the oncologic impact of surgery is not uniform across patient subgroups. These effect modifiers are biologically plausible, given well-documented sex-specific differences in hormonal milieu, adipose tissue distribution, insulin sensitivity, and cancer susceptibility, as well as age-related variation in cancer latency, baseline risk, and competing causes of morbidity and mortality.

Across large observational cohorts, women consistently appear to derive greater reductions in cancer incidence after BS than men. Sjöholm et al. reported a significantly lower overall cancer incidence among women undergoing BS compared with matched controls, whereas no corresponding reduction was observed in men, indicating a clear sex–treatment interaction [201]. Notably, the protective association in women was most pronounced for female-specific and obesity-related cancers, supporting the involvement of endocrine and metabolic pathways in mediating these effects [201].

These observations are further reinforced by pooled evidence. In a meta-analysis of observational studies, Tee et al. reported a significant reduction in overall cancer risk among women after BS (RR 0.68, 95% CI 0.60–0.77), whereas no significant association was observed in men (RR 0.99, 95% CI 0.74–1.32) [193]. Similarly, a large meta-analysis encompassing approximately 18 million individuals found that the inverse association between BS and cancer risk was substantially stronger in women than in men, reinforcing sex as a key modifier of post-surgical cancer risk reduction [202].

Sex-specific heterogeneity is particularly evident for CRC. In a matched analysis including 88,630 surgically treated patients and 327,734 controls with severe obesity, women experienced a significant reduction in CRC risk after BS, driven primarily by RYGB (HR 0.40; 95% CI 0.18–0.87; *p* = 0.02). In contrast, men did not show a comparable benefit and instead exhibited a trend toward increased CRC risk relative to controls, particularly beyond three years of follow-up (OR 2.18, 95% CI 0.97–4.89; *p* = 0.06). Direct sex comparisons further demonstrated a substantially higher post-surgical CRC risk in men than in women, with the excess concentrated in rectosigmoid tumors (HR 2.69; 95% CI 1.35–5.38; *p* < 0.001) [203].

Age at surgery also appears to influence cancer outcomes. In a nationwide Nordic cohort focusing on adults aged ≥60 years, BS as a composite exposure was not consistently associated with a reduction in obesity-related cancer incidence compared with matched nonsurgical controls [204]. However, within this older population, a specific bariatric procedure was associated with a lower risk of obesity-related cancer (HR 0.74; 95% CI 0.56–0.97), suggesting that procedure type and residual life expectancy may interact with age to shape long-term cancer risk trajectories [204].

In line with these age-related differences, a large population-based cohort from Ontario showed that BS was linked to a significant reduction in cancer-related mortality (HR 0.54; 95% CI 0.36–0.80). Subgroup analyses revealed the largest absolute mortality benefit among adults aged ≥55 years, highlighting the potential importance of timing in relation to cancer latency and competing risks [205].

Overall, current evidence indicates that sex and age are key effect modifiers in the relationship between BS and cancer outcomes. Women—particularly those undergoing surgery at younger or midlife ages—consistently demonstrate greater reductions in cancer incidence, whereas effects in men are weaker, heterogeneous, or confined to specific cancer sites. Similarly, the apparent cancer-protective association of BS tends to attenuate with advancing age, likely reflecting shorter latency windows, higher baseline risk, and competing causes of morbidity. These patterns underscore the importance of stratified interpretation of cancer outcomes after BS and caution against extrapolating average effects across populations without accounting for sex, age, and procedural context.

Collectively, the available evidence suggests that BS is associated with lower overall cancer incidence and cancer-related mortality at the population level, particularly for obesity-associated malignancies; however, these associations remain heterogeneous and context dependent. Observed cancer outcomes vary according to study design, comparator selection, duration of follow-up, cancer site, surgical procedure, and patient characteristics such as sex and age at surgery. Persistent site-specific signals, procedure-related differences, and subgroup variability highlight the inherent limitations of observational inference, emphasizing that current data support associative patterns rather than definitive causal conclusions regarding the relationship between BS and long-term cancer risk.

## 5. Nutritional Mechanisms in Cancer Risk Modulation Post-Surgery

BS not only induces substantial and durable weight loss but also reshapes long-term nutritional physiology. These postoperative changes can modulate biological systems involved in carcinogenesis, including immune regulation, endocrine signaling, oxidative balance, and the integrity of cellular repair mechanisms. Thus, the net oncological impact of bariatric procedures depends not only on metabolic improvement but also on the quality of postoperative nutritional management. Evidence from long-term clinical trials such as the 10-year SLEEVEPASS study illustrates how these nutritional alterations differ by surgical technique, particularly in relation to micronutrient status [206].

### 5.1. Micronutrient Deficiencies and Their Potential Role in Carcinogenesis

Micronutrient deficiencies represent one of the most clinically significant and biologically relevant long-term consequences of BS. Recent prospective data show that deficiencies in iron, folate, and vitamin B12 tend to increase with postoperative follow-up, indicating that the nutritional vulnerability of these patients persists—and may even worsen—over time [207]. This temporal pattern is particularly evident after malabsorptive procedures. In the SLEEVEPASS trial, for instance, iron deficiency was substantially more frequent 10 years after RYGB than after SG, despite similar metabolic improvements [206]. Complementary evidence from a network meta-analysis further confirms that RYGB is associated with higher risks of iron, vitamin B12, calcium, and vitamin D deficiencies compared with restrictive techniques [208]. Together, these findings highlight a paradox: although BS improves obesity-related metabolic risk, it also induces persistent micronutrient disturbances. Because these nutrients are essential for DNA synthesis and immune function, their long-term deficiency may plausibly influence cancer susceptibility.

Among postoperative deficiencies, those affecting one-carbon metabolism (vitamin B12 and folate) and vitamin D appear to have the strongest biological plausibility for influencing carcinogenesis, whereas evidence linking iron and calcium disturbances to oncologic risk remains more indirect. This hierarchy provides a useful framework for interpreting the potential relevance of postoperative nutritional alterations in cancer biology.

Iron is central to multiple cellular processes, including mitochondrial respiration and redox balance. Chronic iron deficiency after BS is strongly associated with anemia and reduced cellular function [209]. Long-term evidence from clinical trials illustrates how iron deficiency remains one of the most prevalent postoperative alterations, particularly in bypass procedures [206]. In postoperative cohorts, deficiencies in both iron and vitamin B12 have been directly associated with anemia following RYGB, reinforcing the importance of systematic biochemical monitoring and replacement [210]. Because iron also participates in enzymes involved in DNA repair and oxidative stress defense, its chronic depletion may plausibly contribute to genomic instability in the long term, although direct oncologic evidence remains limited.

Deficiencies in vitamin B12 and folate are especially relevant for carcinogenesis. Both vitamins are essential for one-carbon metabolism, a biochemical network responsible for nucleotide synthesis and DNA methylation. A recent mechanistic review emphasizes that alterations in this pathway are directly implicated in cancer-related epigenetic and genomic processes [211]. From an epidemiological standpoint, a comprehensive meta-analysis of prospective cohorts reported that higher dietary folate intake is associated with reduced CRC risk, supporting the biological importance of maintaining adequate folate levels [211]. The persistence of vitamin B12 deficiency after BS has also been documented in the literature, where differences in adherence to supplementation were closely related to postoperative B12 status [206]. Importantly, recent dose–response meta-analytic evidence shows that elevated circulating vitamin B12 is associated with higher all-cause mortality and provides limited or inconsistent evidence for cancer mortality, reinforcing that postoperative management should focus on restoring physiological levels rather than indiscriminate supplementation [212].

Vitamin D deficiency is another highly prevalent and clinically relevant issue after BS. Beyond its classical role in bone metabolism, vitamin D participates in cellular differentiation, apoptosis, and immune modulation. A 2024 umbrella review reported that higher circulating 25-hydroxyvitamin D concentrations are generally associated with lower cancer incidence and mortality across multiple cancer sites, although the evidence remains primarily observational [174]. In long-term bariatric cohorts, vitamin D insufficiency persisted even a decade after surgery, particularly among patients with low adherence to supplementation [206]. Taken together, mechanistic and epidemiological signals position vitamin D as one of the micronutrients with the most consistent—albeit non-causal—links to cancer-related outcomes.

Calcium homeostasis is closely linked to vitamin D status and is also affected by malabsorptive bariatric procedures. In large European prospective cohorts, prediagnostic serum calcium concentrations have been associated with CRC risk [213]. This is particularly relevant in bariatric populations, where impaired calcium absorption may coexist with vitamin D deficiency, potentially influencing not only skeletal integrity but also epithelial and metabolic pathways related to carcinogenesis. Long-term clinical data confirm that hypocalcemia can occur postoperatively, albeit at relatively low frequency when supplementation is maintained [206].

Overall, current evidence suggests that postoperative micronutrient deficiencies differ in their oncologic relevance. Alterations affecting one-carbon metabolism and vitamin D pathways present the most coherent mechanistic links to carcinogenesis, whereas the contributions of iron and calcium are more likely to be indirect or context dependent. Importantly, the relationship between micronutrient status and cancer risk is supported primarily by mechanistic and observational data rather than definitive causal inference.

### 5.2. Protein and Energy Balance: Implications for Sarcopenia, Cachexia, and Immune Function

Post-bariatric physiology reshapes energy intake and macronutrient handling in ways that can meaningfully influence lean-tissue biology. While intentional weight loss is expected, the quality of weight loss—specifically, how much fat-free mass is preserved—matters because skeletal muscle is tightly linked to metabolic resilience and immune competence, both of which are relevant to cancer susceptibility and clinical outcomes.

Body composition studies consistently show that BS reduces fat-free mass alongside fat mass, particularly during the rapid weight-loss phase. Detailed post-RYGB phenotyping demonstrates measurable declines in lean mass and muscle-related parameters after surgery, highlighting that muscle preservation is not guaranteed without targeted nutritional and lifestyle strategies [214]. Longer-term trajectories, however, suggest substantial heterogeneity. In some cohorts, fat-free mass and skeletal muscle are relatively maintained between years 1 and 5, implying that the early post-surgical period may represent the most vulnerable window for clinically relevant lean-mass losses, while subsequent stabilization is possible with adequate follow-up [215]. At the evidence-synthesis level, a meta-analysis focusing on lean mass and handgrip strength reported that BS is associated with lean mass loss, whereas strength changes are less predictable—supporting the concept that functional decline is not inevitable but modified by diet quality and physical activity [216].

This becomes oncologically relevant because sarcopenia is repeatedly associated with adverse cancer outcomes across multiple settings. Contemporary oncology evidence consistently links sarcopenia to higher mortality and poorer treatment tolerance, reinforcing the importance of lean-mass preservation in long-term cancer risk and survivorship frameworks [217].

Protein intake is a central determinant of lean-mass preservation after BS, yet achieving adequate intake is frequently challenging due to early satiety, food intolerance, and reduced intake capacity. A systematic review assessing protein supplementation after SG found that supplementation significantly improved biochemical markers of nutritional status—including total protein, albumin, magnesium, and iron—reflecting improved protein adequacy rather than isolated micronutrient effects [218]. However, although supplemented patients showed a tendency toward better maintenance of skeletal muscle mass, this effect did not reach statistical significance, indicating that protein supplementation alone may be insufficient to fully prevent postoperative muscle loss [219]. These findings suggest that biochemical repletion and structural muscle preservation are related but not equivalent outcomes.

Intervention trials support a combined strategy. A randomized trial demonstrated that resistance training combined with additional protein intake can mitigate declines in muscle strength after BS, indicating that lean-tissue vulnerability is modifiable when adequate anabolic stimuli are provided [220]. In parallel, systematic evidence indicates that structured exercise training before and after BS improves physical fitness and functional outcomes, strengthening the rationale for embedding exercise into long-term postoperative care when muscle preservation is a clinical priority [221].

Post-bariatric changes also extend to immune regulation. A study comparing immune and metabolic changes after SG and RYGB reported that weight loss was accompanied by shifts in circulating T-cell populations, including reduced activated CD8^+^ cytotoxic T cells and increased regulatory T cells, resulting in a more anti-inflammatory CD4^+^/CD8^+^ balance [222]. These findings suggest that BS may partially restore immune homeostasis by reversing obesity-related immune dysregulation, a process with plausible relevance for long-term cancer risk modulation.

Given that inadequate protein and energy availability can impair immune-cell production and function, postoperative protein–energy balance emerges as a plausible upstream determinant of immune surveillance capacity. Although not specific to bariatric populations, broader clinical nutrition literature consistently shows that malnutrition is associated with impaired immune function and higher infection risk. This supports the biological rationale for prioritizing adequate protein and energy intake during both recovery and long-term follow-up after BS [223].

### 5.3. Alcohol Metabolism After Surgery and Its Role in Upper Gastrointestinal Cancer Risk

Bariatric procedures, especially RYGB, can fundamentally change ethanol pharmacokinetics, leading to faster absorption and higher peak blood alcohol concentrations (BAC) after the same alcohol dose. Classic controlled data show a markedly shorter time-to-peak and higher peak ethanol levels in bypass patients versus controls, consistent with accelerated delivery of ethanol to the small intestine and reduced buffering by the stomach [224]. More recent work measuring early post-ingestion time points confirms that, after RYGB, BAC can rise rapidly within minutes, reaching levels that may not be captured by standard breathalyzer timing protocols [225].

Mechanistically, several factors likely converge. These include rapid gastric pouch emptying with accelerated intestinal delivery of ethanol and reduced first-pass metabolism in the stomach due to altered anatomy and potentially lower gastric alcohol dehydrogenase activity, both of which increase systemic exposure for a given intake [225]. Clinically, these changes are relevant because they can unintentionally shift drinking patterns, such that modest intake produces disproportionately high BAC, increasing cumulative exposure to ethanol and its metabolites over time.

Evidence for SG is more heterogeneous than for RYGB, partly reflecting methodological differences across studies. Work using gas chromatography—the gold standard for BAC measurement—indicates that SG, similar to RYGB, can result in faster absorption and higher peak BAC than pre-surgery comparators, while also demonstrating that breath alcohol measurements may underestimate true peak exposure [226]. However, other investigations report minimal change in alcohol metabolism after SG, underscoring the influence of surgical technique, anatomical variation, and measurement approach [227].

These pharmacokinetic changes are relevant to cancer risk because ethanol’s carcinogenicity is largely mediated by acetaldehyde, its first metabolite [228]. While the broader carcinogenic mechanisms of alcohol are well established, the upper aerodigestive tract—particularly the esophagus—is uniquely vulnerable, as acetaldehyde can be generated locally and directly contact mucosal surfaces [229,230,231]. In this context, a post-bariatric shift toward higher peak ethanol exposure may plausibly increase mucosal acetaldehyde burden per drinking episode, compounding epithelial injury and DNA damage risk across repeated exposures [232].

At the population level, alcohol intake is consistently associated with increased OEC risk, particularly esophageal squamous cell carcinoma (ESCC). A dose–response meta-analysis reports that alcohol consumption significantly increases OEC incidence, with stronger associations for ESCC than for adenocarcinoma in many datasets [161]. Earlier pooled evidence similarly concluded that alcohol intake above guideline levels markedly increases ESCC risk, with smoking modifying risk in some analyses [233]. Together, these findings support the view that any post-surgical state facilitating higher effective ethanol exposure per drink could be relevant to upper gastrointestinal cancer risk, particularly for ESCC and in the presence of co-exposures such as tobacco or reflux-related injury.

Importantly, BS is also associated with a non-trivial risk of new-onset alcohol misuse or alcohol use disorder, with the most consistent signals observed after RYGB and emerging evidence after SG. A recent systematic review supports consensus guidance that patients should be informed about this medium- to long-term risk, reinforcing the need for anticipatory counseling and follow-up [234]. This behavioral dimension is critical, as cancer risk reflects cumulative exposure over time, and altered pharmacokinetics combined with increased intake frequency or quantity can substantially increase lifetime ethanol and acetaldehyde burden.

Taken together, these pharmacokinetic and behavioral factors provide a biologically plausible pathway linking BS-associated alcohol vulnerability to upper gastrointestinal cancer risk. This is particularly relevant after RYGB, where disproportionately high BAC peaks and acetaldehyde exposure may occur even with modest alcohol intake, underscoring the importance of patient awareness regarding potential carcinogenic exposure in the postoperative setting [232].

### 5.4. Gut Microbiome Changes After Surgery and Their Possible Role in Tumorigenesis

BS also produces sustained remodeling of the gut microbiome that extends beyond short-term dietary shifts [32]. In a controlled human study comparing bariatric interventions, RYGB produced the largest microbiota shifts and was associated with increased microbial diversity and enrichment of taxa considered metabolically beneficial (e.g., *Faecalibacterium prausnitzii*), suggesting that surgery can reset dysbiosis beyond what is expected from weight loss alone [235]. Importantly, long-term follow-up indicates that these microbiome differences can persist for up to a decade after surgery, supporting a sustained biological exposure rather than a transient postoperative phenomenon [236].

However, cancer relevance depends less on which taxa change and more on how microbial function and metabolite production are altered. Multi-omics human studies consistently demonstrate that BS reshapes not only microbial composition but also fecal metabolic outputs, supporting the view that any post-surgical tumorigenic signaling—if present—would most plausibly be mediated through altered luminal metabolites and host–microbe metabolic crosstalk rather than microbiota composition alone [237,238].

A key mechanistic axis involves BA metabolism, which is tightly microbiome-dependent and strongly modified by bariatric anatomy through changes in nutrient flow, intestinal transit, and enterohepatic cycling. Recent human data explicitly link BS to alterations in secondary BA profiles and microbial BA-metabolism capacity in ways framed as relevant to colorectal carcinogenesis. A 2024 pilot study reported lower fecal lithocholic acid concentrations and reduced abundance of bacterial genes required for BA metabolism after BS, directly discussing these changes as potential modulators of the CRC cascade [239].

Beyond BAs, emerging evidence suggests that specific microbiota-associated metabolites after RYGB may exert pro-tumorigenic effects. A 2025 open-access study reported that RYGB-associated fecal bacteria could contribute to increased tyramine production, and that tyramine exposure increased CRC risk through enhanced DNA damage, increased epithelial proliferation, and pro-inflammatory gut responses. This provides a concrete metabolite-based mechanism linking post-surgical microbial output to tumorigenic pathways [240].

Human colorectal biomarker studies yield mixed findings. Earlier work reported increased colorectal epithelial proliferation and crypt fission in association with obesity and RYGB, raising concerns that some post-RYGB intestinal adaptations could align with tumorigenic potential [241]. By contrast, subsequent biomarker-focused clinical studies observed reduced rectal crypt cell proliferation and lower inflammatory marker expression at specific postoperative time points, particularly at 6 months post-RYGB, and interpreted these patterns as consistent with decreased tumorigenic potential [241].

**Figure 2:**
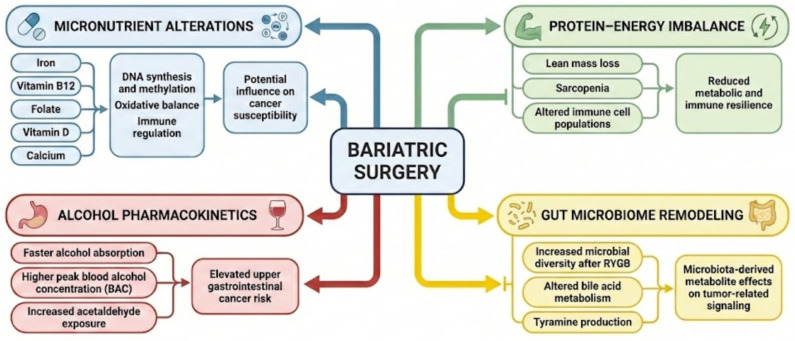
Long-term Nutritional and Biological Mechanisms of BS Modulating Cancer Risk. Created in BioRender. Reytor, C. (2026) https://BioRender.com/j31kgzj.

Taken together, the most defensible interpretation is that BS induces long-lasting microbiome remodeling [236], but its implications for tumorigenesis are context dependent. Relevant modifiers include procedure type, timing after surgery, and—critically—the metabolite profile generated by the remodeled microbiome. The strongest emerging evidence for tumor-related signaling is currently anchored in metabolite-level changes (e.g., BA and tyramine) rather than taxonomic descriptions alone [239,240] (Figure 2).

These microbiome-driven effects intersect with postoperative nutritional exposures rather than operating independently. The magnitude and persistence of metabolite shifts vary by surgical technique and are strongly modified by postoperative dietary quality, which conditions epithelial integrity, immune competence, and luminal metabolite exposure over time [206]. Diet quality, therefore, emerges as a primary modulator of the post-surgical microbial–metabolic environment, influencing whether surgery-associated microbiome remodeling yields a favorable or potentially adverse metabolite profile with plausible implications for tumor-related signaling [237].

Accordingly, expert guidance supports structured biochemical surveillance and individualized nutritional counseling as essential components of long-term postoperative care [242]. In this framework, sustained follow-up is not merely supportive care but a determinant of the durability of metabolic—and potentially oncologic—benefits after BS. Evidence from long-term cohorts reinforces this view, highlighting postoperative nutritional management as a modifiable exposure capable of shaping long-term metabolic and oncologic trajectories [243].

Taken together, BS induces durable microbiome remodeling with measurable metabolic consequences; however, whether these alterations translate into clinically meaningful cancer risk modification remains uncertain. Current evidence is largely mechanistic and associative, and long-term prospective human studies are needed to determine if post-bariatric microbial and metabolite shifts exert a causal influence on tumorigenesis.

BS influences four interconnected pathways: micronutrient deficiencies affecting DNA repair and immune regulation [206,207,208]; protein–energy imbalance associated with sarcopenia and reduced metabolic resilience [214,215,216]; altered alcohol metabolism increasing systemic ethanol and acetaldehyde exposure [229,230,231]; and gut microbiome remodeling that changes BA and metabolite signaling [237,238]. These processes collectively shape the long-term oncological impact of surgery. Abbreviations: BAC: blood alcohol concentration; RYGB: Roux-en-Y Gastric Band.

## 6. Clinical Implications for Long-Term Nutritional Management

Clinical translation of these mechanisms requires treating postoperative nutrition as a longitudinal, risk-based exposure, not as isolated deficiencies corrected episodically. From a cancer-prevention perspective, nutritional management after BS should be prioritized around a small number of high-impact, modifiable strategies rather than diffuse, non-specific recommendations [244]. Contemporary guidance emphasizes structured multidisciplinary preoperative assessment, including dietitian-led evaluation to identify maladaptive eating patterns, optimize baseline nutritional status, and prepare patients for long-term dietary change, with procedure selection and follow-up intensity informed by differential nutritional risk across techniques [245,246]. Preoperative screening should explicitly address that micronutrient deficiencies are common even before surgery, strengthening the rationale for baseline labs and targeted correction rather than assuming deficiencies are purely postoperative [169]. In parallel, cancer prevention should be operationalized pragmatically, ensuring that guideline-appropriate cancer screening is up to date before surgery, particularly in individuals with elevated baseline risk or obesity-related screening barriers [247].

Postoperatively, supplementation and biochemical monitoring should be individualized according to the type of bariatric procedure, as malabsorptive techniques confer a greater long-term risk of deficiencies than restrictive surgeries [246]. In this context, the objective is to maintain physiological micronutrient levels through targeted repletion and continuous surveillance, rather than empiric or non-specific supplementation (Table 3). Consensus guidance recommends routine daily multivitamin–mineral supplementation containing thiamine, iron, zinc, copper, selenium, and folate for all procedures, with additional micronutrients as indicated by the American Society for Metabolic and Bariatric Surgery guidelines [22]. Calcium citrate (1200–1500 mg/day) combined with vitamin D is recommended to support bone health and may require adjustment based on 25(OH)D levels and parathyroid hormone results [242]. For iron, supplemental elemental iron (often 100–200 mg/day) is advisable after RYGB and SG, with co-administration of vitamin C to enhance absorption when appropriate [21,242]. Vitamin B12 deficiency often requires routine supplementation, with intramuscular injections (e.g., 1000 µg every 3 months) in patients with impaired absorption [21]. Routine biochemical surveillance at 3–6 months post-surgery and annually thereafter aids early detection and correction of emerging deficiencies [21,247].

Among all postoperative dietary priorities, adequate protein intake should be treated as a first-order target. Current bariatric nutrition guidance suggests protein intake goals after surgery of ≥60–80 g/day or 1.0–1.5 g/kg of ideal body weight, with higher targets considered after more extensive or malabsorptive procedures [248,249]. Failure to meet minimum protein thresholds is common in routine practice and represents a modifiable risk factor for sarcopenia, immune impairment, and adverse long-term outcomes [250]. Accordingly, early dietary planning, use of protein supplementation when needed, and reinforcement during follow-up visits should be prioritized over less impactful dietary refinements [251,252,253].

Beyond protein quantity, overall diet quality is a key modulator of postoperative metabolic and inflammatory trajectories. Dietary patterns emphasizing nutrient-dense foods—such as legumes, lean proteins, eggs, dairy, fruits, vegetables, and whole grains—support both weight management and micronutrient adequacy [254]. Patterns resembling a Mediterranean-style diet are associated with greater total weight loss and may favorably influence microbiome composition and metabolite profiles after BS [255,256,257]. Such patterns are also aligned with reduced chronic inflammation and improved metabolic profiles linked to lower long-term cancer risk, supporting their prioritization in postoperative counseling [258,259,260].

Conversely, a reduction in UPF should be an explicit counseling target rather than an implicit recommendation. UPF intake is associated with poorer nutrient density, adverse metabolic profiles, and higher cancer risk in the general population, and may undermine the metabolic and inflammatory benefits of BS if not addressed directly [149,261]. While very low-carbohydrate ketogenic diets may have a role as time-limited, targeted interventions for weight regain under specialist supervision, they should not replace balanced, nutrient-dense dietary patterns as a long-term strategy [262].

Alcohol intake warrants dedicated counseling, particularly after RYGB. Given altered alcohol pharmacokinetics and the increased risk of alcohol misuse after surgery, patients should be counseled pre- and postoperatively about heightened intoxication per drink and potential long-term risks, including upper gastrointestinal cancer susceptibility [27,234,263]. Alcohol counseling should therefore be integrated into routine postoperative follow-up rather than addressed reactively.

Micronutrient supplementation should balance adequacy with caution. While correction of deficiencies is essential, excessive or non-indicated supplementation—particularly of folate and vitamin B12—should be avoided in the absence of documented deficiency, given emerging concerns about potential pro-tumorigenic signaling in specific contexts [264,265,266]. This reinforces the importance of laboratory-guided, individualized supplementation strategies rather than blanket dosing. Key clinical takeaways are summarized in Box 1.

Taken together, these considerations underscore that BS should be regarded as a long-term nutritional and oncologic management process rather than solely a metabolic intervention [267,268]. Optimal care requires integration of bariatric teams, clinical nutrition, and oncology-informed risk management, ensuring that nutritional surveillance, behavioral counseling, alcohol risk assessment, and preventive screening are continuous and coordinated rather than fragmented [245,246,247,268].

**Table 3:** Recommended long-term nutritional management after BS.

Component	General Recommendation	Procedure-Specific Notes	Monitoring	Key References
Multivitamin–mineral	Daily, includes thiamine, iron, zinc, copper, selenium, folate	Higher vigilance after RYGB/BPD-DS	Baseline, 3–6 mo, annually	[22]
Iron	100–200 mg elemental/day + vitamin C	Higher doses for menstruating women and RYGB	Ferritin, Hb every 6–12 mo	[21,242]
Vitamin B12	350–500 µg/day oral or 1000 µg IM q3 mo	IM preferred after RYGB or low adherence	Serum B12 annually	[21]
Calcium + Vitamin D	1200–1500 mg Ca citrate + 3000 IU vit D3/day	Adjust by PTH and 25(OH)D	Vit D and PTH annually	[242]
Protein intake	≥60–80 g/day or 1.0–1.5 g/kg ideal body weight	Higher targets after malabsorptive surgery	Diet recall at follow-up	[248,249,250]
Dietary Pattern	Nutrient-dense, Mediterranean-style	VLCKD only if supervised and short-term	Clinical and metabolic follow-up	[254,255,256,262]
Alcohol counseling	Routine screening and education	Greater sensitivity after RYGB	Behavioral follow-up	[22]

Abbreviations: BS: Bariatric surgery; RYGB: Roux-en-Y gastric bypass; BPD-DS: Biliopancreatic diversion with duodenal switch; IM: Intramuscular; Hb: Hemoglobin; PTH: Parathyroid hormone; VLCKD: Very low-calorie ketogenic diet.

Box 1 **Key Clinical Takeaways for Cancer Risk Mitigation After BS.**

- BS should be conceptualized as a long-term metabolic and oncologic exposure rather than a single weight-loss intervention.
- Sustained nutritional surveillance is a core component of post-BS care, as persistent micronutrient deficiencies—particularly those affecting vitamin D and one-carbon metabolism (folate/B12)—may influence pathways linked to carcinogenesis.
- Overall diet quality remains a major modifier of postoperative inflammatory and metabolic trajectories associated with long-term cancer risk.
- Altered alcohol pharmacokinetics after RYGB may increase effective carcinogenic exposure per drinking episode, supporting the relevance of anticipatory clinical counseling.
- Long-term risk mitigation is supported by structured follow-up integrating nutritional monitoring, behavioral assessment, and preventive care.

## 7. Conclusions

BS represents one of the most effective long-term interventions for severe obesity and is increasingly recognized for its potential to modify cancer risk. Across large observational cohorts and recent meta-analyses, BS is consistently associated with lower overall cancer incidence and cancer-related mortality, particularly among women and for obesity-related cancers such as postmenopausal breast, endometrial, colorectal, and pancreatic malignancies. However, this protective effect is neither uniform nor unconditional. The same physiological alterations that improve metabolic health also impose persistent nutritional vulnerabilities that can influence long-term biological resilience.

Micronutrient deficiencies, protein–energy imbalance, altered alcohol metabolism, and surgery-induced microbiome remodeling emerge as central determinants of the postoperative carcinogenic environment. These factors are not merely secondary effects of surgery; they actively interact with endocrine, immune, inflammatory, and metabolic pathways known to be involved in tumorigenesis. Consequently, the long-term oncological benefit of BS depends not only on the magnitude of weight loss but also on how effectively nutritional health is preserved and managed after surgery.

Sustained, individualized nutritional care should therefore be considered a cornerstone of both metabolic and oncological outcomes in post-bariatric patients. Multidisciplinary strategies that integrate bariatric teams, clinical nutrition, and oncology-informed risk management are essential to ensure adequate surveillance, tailored supplementation, and evidence-based dietary counseling. These approaches can help mitigate nutritional deficiencies while supporting the favorable metabolic and immunologic changes that underpin the observed reductions in cancer risk.

Finally, the current evidence base remains predominantly observational, underscoring the urgent need for well-designed, long-term prospective studies that directly evaluate how postoperative nutritional exposures influence cancer incidence and mortality. Clarifying these relationships will be critical for translating BS from a purely metabolic therapy into a more comprehensive tool for cancer prevention and long-term health optimization.

## Figures and Tables

**Table 1:** Biological Pathways Linking Obesity to Major Cancer Types.

Cancer Site	Dominant Pathways in Obesity	Consistency of Human Evidence *
Colorectal cancer [144,145,146,147,148,149,150,151,152,153,154,155,156]	↑ insulin–IGF; ↑ mucosal inflammation; ↑ secondary BAs; ↓ gut barrier	●●●
Breast cancer [14,38,42,46,47,69,70,71,72,73,74,78,79,118,133,135,136,137,142,157,158]	↑ estrogen (↑ aromatase, ↓ SHBG); ↑ IR; ↑ leptin ↓ adiponectin; ↑ inflammation	●●
Endometrial cancer [38,85,88,89,90,91,132,158]	↑ unopposed estrogen; ↑ IR; ↑ inflammation	●●
Pancreatic cancer [49,50,51,61,67,107,108,110]	↑ hyperinsulinemia; ↑ metabolic inflammation; ↑ mTOR signaling	●●
Hepatocellular carcinoma [103,124,125,159]	↑ MASLD/MASH injury; ↑ IL-6/TNF-α → STAT3; ↑ gut–liver axis	●●
Gastric cancer (non-cardia) [109,145,146,147,149,160]	↑ chronic inflammation; ↑ metabolic dysregulation; microbiota-related mechanisms	●
Esophageal adenocarcinoma [146,147,149,161]	↑ central adiposity—GERD; ↑ inflammation; ↑ insulin–IGF	●

Abbreviations: ↑ increase; ↓ decrease; IR, insulin resistance; IGF, insulin-like growth factor; BAs, bile acids; mTOR, mechanistic target of rapamycin; SHBG, sex hormone binding globulin; MASLD/MASH, metabolic dysfunction-associated steatotic liver disease/steatohepatitis; GERD, gastroesophageal reflux disease. Consistency of human evidence: ●●● = consistent findings across large prospective cohorts and metaanalyses; ●● = multiple prospective cohorts and/or pooled analyses with generally concordant results but some heterogeneity; ● = limited or emerging human evidence, often site-specific or supported by fewer prospective studies. * This column reflects a qualitative synthesis of the human evidence cited in this review; no formal grading system was applied, and symbols should not be interpreted as quantitative measures of causal strength.

**Table 2:** Dietary Exposures and Nutrient Imbalances Associated with Obesity-Related Cancers.

Cancer Site	Dietary Factors	Nutrient Imbalances
Colorectal cancer [144,145,146,147,148,149,150,151,152,153,154,155,156]	UPF; processed/red meat; ↓ fiber/wholegrains; alcohol; ↑ DII	↓ vitamin D; ↓ folate/B12; iron dysregulation
Breast cancer [14,38,42,46,47,69,70,71,72,73,74,78,79,118,133,135,136,137,142,157,158]	Alcohol; UPF-rich patterns; low diet quality	↓ vitamin D; ↓ folate/B12
Endometrial cancer [38,85,88,89,90,91,132,158]	Pro-IR/pro-inflammatory dietary patterns	↓ vitamin D; ↓ folate/B12; ↓ iron
Pancreatic cancer [49,50,51,61,67,107,108,110]	High glycemic load; ↓ fiber (food-specific signals inconsistent)	↓ vitamin D; ↓ folate/B12
Hepatocellular carcinoma [103,124,125,159]	Alcohol; energy-dense/low-quality patterns	↓ vitamin D; iron dysregulation
Gastric cancer (non-cardia) [109,145,146,147,149,160]	UPF; high-salt/processed patterns	↓ iron; ↓ folate/B12
Esophageal adenocarcinoma [146,147,149,161]	Energy-dense patterns (reflux-related); alcohol	↓ vitamin D (indirect)

Abbreviations: ↑ increase; ↓ decrease; IR, insulin resistance; UPF, ultra-processed foods; DII, Dietary Inflammatory Index.

## Data Availability

No new data were created or analyzed in this study. Data sharing is not applicable to this article.

## Abbreviations

AGB	Adjustable gastric banding
BS	Bariatric surgery
BA	Bile acid
BPD-DS	Biliopancreatic diversion with duodenal switch
BAC	Blood alcohol concentrations
BMI	Body mass index
BC	Breast cancer
CRC	Colorectal cancer
EOCRC	Early-onset colorectal cancer
EC	Endometrial cancer
OEC	Esophageal cancer
ECM	Extracellular matrix
HR	Hazard ratio
HCC	Hepatocellular carcinoma
IR	Insulin resistance
IGF	Insulin-like growth factor
IL-6	Interleukin-6
IARC	International Agency for Research on Cancer
JAK/STAT3	Janus kinase/signal transducer and activator of transcription 3
mTOR	Mechanistic target of rapamycin
mTORC1	Mechanistic target of rapamycin complex 1
MR	Mendelian randomization
NK	Natural killer
TNF-α	Necrosis factor alpha
NF-κB	Nuclear factor kappa B
ODDS	Obesity and Disease Development Sweden
PC	Pancreatic cancer
PI3K	Phosphoinositide 3-kinase
AKT	Protein kinase B
RYGB	Roux-en-Y gastric bypass
SHBG	Sex hormone–binding globulin
SCFAs	Short-chain fatty acids
SG	Sleeve gastrectomy
TME	Tumor microenvironment
UPF	Ultra-processed food
VEGF	Vascular endothelial growth factor
WHR	Waist-to-hip ratio
